# Molecular Characterisation, Isolation, and Antibody Response to Influenza D Virus in Naturally Infected Cattle

**DOI:** 10.3390/v18060626

**Published:** 2026-05-29

**Authors:** Hasan Emre Tali, Huseyin Yilmaz, Gizem Karadag, Nuri Turan, Ana Moreno, Mariette F. Ducatez, Juergen A. Richt, Aysun Yilmaz

**Affiliations:** 1 Department of Virology, Veterinary Faculty, Istanbul University-Cerrahpasa, Hadimkoy, Istanbul 34500, Türkiyehyilmaz@iuc.edu.tr (H.Y.);; 2 Istituto Zooprofilattico Sperimentale Della Lombardia e dell’Emilia Romagna, 25124 Brescia, Italy; 3 Interactions Hôtes-Agents Pathogènes (IHAP), University of Toulouse, INRAE, ENVT, 23 Chemin des Capelles, P.O. Box 87614, 31076 Toulouse CEDEX 3, France; 4 Department of Diagnostic Medicine and Pathobiology, College of Veterinary Medicine, Kansas State University, Manhattan, NY 66506, USA; 5 Department of Veterinary Tropical Diseases, Faculty of Veterinary Science, University of Pretoria, Onderstepoort 0110, South Africa

**Keywords:** influenza D virus, cattle, PCR, ELISA, HI, Türkiye

## Abstract

Influenza D virus (IDV) is a respiratory pathogen affecting the health of cattle, thereby causing economic losses. This study was conducted to detect and isolate IDV, investigate its molecular characteristics, and examine the antibody response to IDV in naturally infected cattle. Real-time RT-PCR was used to analyse 279 nasal swabs and blood sera collected from cattle on farms in the Thrace district of Türkiye, bordering the European Union, for IDV-RNA. One sample from 2020 from a small, family-owned farm (D/OK lineage), two samples from 2021 from an integrated farm (D/OK lineage), and two samples from 2024 (D/Yama2019) from an integrated farm were found to be positive for IDV-RNA. All five samples were collected from farms located in Kırklareli. Positive samples were subjected to virus isolation and phylogenetic analyses. Serum samples were analysed using ELISA and HI tests. Of the 279 samples collected, 1.79% tested positive for IDV-RNA in real-time RT-PCR. Seroprevalence, measured for the first time in Türkiye, was found to be 45.5% and 55.19% by ELISA and HI tests, respectively. Key factors statistically associated with IDV seropositivity included the study year, geographical location, and farm type. Clinically, nasal discharge and cough were the primary symptoms in seropositive animals. Genetic analysis revealed that the 2020–2021 samples clustered within the D/OK lineage, while the 2024 samples belonged to the D/Yama2019 lineage. The findings suggest the co-circulation of multiple IDV strains, although this should be interpreted cautiously given the limited number of sequences in the Thrace region. Considering the lack of an influenza D virus (IDV) vaccine, these findings emphasise the importance of continued surveillance and diagnostic testing of cattle to limit viral spread, protect cattle health, and reduce economic losses associated with IDV.

## 1. Introduction

Influenza viruses are divided into four types: A, B, C, and D. IDV is an enveloped, single-stranded, negative-sense RNA virus classified in the *Deltainfluenzavirus* genus, *Orthomyxoviridae* family. The genome consists of seven genomic segments which encode nine proteins: hemagglutinin esterase fusion (HEF) glycoprotein; PB1, PB2, and P3 polymerases; nucleoprotein; matrix proteins (M1 and CM2); and non-structural proteins (NS1 and NEP) [1,2,3]. The size of the segments varies between 0.76 and 2.32 kb; together, they make up the 10 Kb total size of the genome.

IDV was first identified in pigs with respiratory disorders and has subsequently been reported in healthy and diseased cattle in various countries around the world [1,3,4,5,6]. Cattle are the main reservoir for IDV. Young, weaned calves with weak immune systems are the most susceptible group, and this is attributed to the decrease in maternal antibody levels after 4–6 months of age [7]. Clinically, animals infected with IDV show symptoms like dry cough, rhinorrhoea, and respiratory system discomfort [2,4]. The disease may also proceed asymptomatically, but infected animals continue to shed the virus during this period [2,8].

When IDV infection occurs alone in cattle, it is generally not fatal unless the animal has a weak immune system. If it is mixed with other respiratory system pathogens, or if IDV is established first and then facilitates the entry of other pathogens, it results in bovine respiratory disease complex (BRDC) [9]. It has been reported that mortality increases as a result of BRDC caused by Bovine Herpesvirus 1 (BHV-1), Bovine Viral Diarrhea Virus (BVDV), Bovine Respiratory Syncytial Virus (BRSV), Bovine Parainfluenza Virus 3 (BPIV-3), and Bovine Adenovirus 3 (BAV-3); *Mannheimia haemolytica*; *Pasteurella multocida*; *Histophilus somni*; *Micrococcus* spp.; and various stress factors [10,11].

Virus isolation and molecular and serological methods are frequently used to diagnose IDV infections in cattle [5,6,8,12]. IDV has been isolated from various cell lines such as Human Rectal Tumour (HRT-18), Swine Testicle (ST), Madin–Darby Canine Kidney (MDCK), and Human Lung Adenocarcinoma (A549), and the cell lines frequently used in IDV isolation are HRT-18 and ST cells [13,14,15]. The most used tests to analyse antibody response are hemagglutination inhibition, ELISA, and virus neutralisation. Among molecular methods, RT-PCR and real-time RT-PCR are widely used to detect IDV-RNA. In samples found positive with real-time RT-PCR, sequencing is performed with by amplifying the hemagglutinin esterase fusion (HEF) gene of IDV via RT-PCR. With sequencing data, phylogenetic analyses can be performed to determine the IDV genotypes circulating in animal populations [5,8].

Phylogenetic analyses based on the HEF gene have indicated that IDV clusters into five different lineages. D/OK has been detected in Europe (France, Italy, Ireland, Denmark, Spain), America (USA and Mexico), Asia (China), and Africa (Namibia), while D/660 has been reported in Europe (Italy, Denmark, Spain) and America (USA, Canada and Mexico). D/Yama2016 has been detected in Asia (Japan), and D/Yama2019 has been found in Asia (Japan and China) and Türkiye. D/CA2019 has been reported in the United States (USA) [8,16,17,18,19].

BRDC causes major economic losses in the cattle industry worldwide [4]. Increased understanding of the role of IDV in BRDC emphasises the need for an effective and safe vaccine against IDV infection in cattle. At present, there is no specific, effective, and safe IDV vaccine suitable for cattle, and few studies have examined the immune response to natural IDV infection [4,20]. Therefore, the role of IDV in BRDC, circulating genotypes, and the immune response in naturally infected cattle needs to be investigated to mitigate bovine IDV infections. In this study, molecular characterisation, virus isolation, and serological analyses were performed to investigate circulating genotypes and antibody response to IDV infection in cattle.

## 2. Materials and Methods

### 2.1. Study Design and Sample Collection

The purposes of this study were to detect, isolate, and molecularly characterise IDV and investigate the immune response to IDV in naturally infected cattle in the Thrace district in Türkiye, which borders the EU. This region has both integrated and family-owned cattle farms producing beef and dairy products. There are around 488.000 cattle in the Thrace district [21]. Cattle diseases in this region are of the utmost importance due to the import of cattle and cattle products, as well as cattle movements in terms of the transboundary transmission of animal diseases. The study area comprises the Thrace region of Türkiye, located in the European part of the country, and includes the provinces of Edirne, Kırklareli, Tekirdağ, and the European side of Istanbul. This region is bordered by Greece and Bulgaria to the west and northwest and by the Black Sea and the Sea of Marmara to the north and south, respectively [22]. Climatically, Thrace exhibits a transitional regime between Mediterranean and continental climates. Mean annual temperatures range between 12 and 15 °C, with summer temperatures often exceeding 30 °C and winter temperatures occasionally dropping below 0 °C. Annual precipitation varies between 500 and 800 mm, with higher rainfall in the northern, elevated areas and along the Black Sea coast. Land in Thrace is predominantly used for agriculture and livestock production. Intensive animal farming, particularly of cattle and small ruminants, is very common [23].

In this study, integrated and small, family-owned cattle farms in Kırklareli, Edirne, Tekirdağ, and Istanbul in the Thrace district were visited. Nasal swabs and blood samples were collected from animals showing respiratory symptoms. A total of 19 farms consisting of 9 small, family-owned and 10 integrated farms were visited between February 2020 and April 2024, and 279 nasal swabs (duplicates) and blood samples were collected from day-old to 12-month-old cattle with respiratory symptoms such as dyspnoea, nasal discharge, coughing, fatigue, and fever. Nasal swabs were taken in duplicate, one of which was placed in a tube containing RNALater stabilisation solution (Invitrogen, Thermo Fisher Scientific, Waltham, MA, USA Cat. No: AM7020) for RT-PCR, while the other was placed in transport medium (Gibco, Thermo Fisher Scientific, Waltham, MA, USA, Cat. No: 11095080) for virus isolation. Blood samples were collected in plain blood tubes (BD Vacutainer, Becton, Dickinson and Company, Franklin Lakes, NJ, USA, Cat. No: 367896) for serological tests. All samples were transported to the laboratory at 4–8 °C.

### 2.2. RNA Extraction and Reverse Transcription

Viral RNA was extracted from the swabs using a PureLink RNA Mini Kit (Invitrogen, Cat. No: 12183025) according to the manufacturer’s instructions. Complementary DNA (cDNA) was generated from the RNA extracts using a High-Capacity cDNA Reverse Transcription Kit (Applied Biosystems, Foster City, CA, USA, Cat. No: 4368814). cDNA was either used directly or kept at −20 °C until required.

### 2.3. Detection of IDV-RNA by Real-Time RT-PCR

All swabs were analysed by probe-based real-time RT-PCR using the primers targeting the polymerase basic 1 (*PB1*) gene of IDV, using the method reported by Faccini et al. (2017) [24] (Table 1). Primers and probes were positioned in the IDV PB1 gene using SnapGene (Version 7.2, IUC, Department of Virology, Istanbul, Türkiye) (Table 1). Briefly, a total volume of 25 μL of PCR mixture per sample, consisting of 12.5 μL of FluoCycle II Probe Master Mix (Euroclone, Pero (MI), Italy, Cat. No: ERD001100BIM), 5 μL of cDNA, 0.75 μL of IDV F primer (10 μM), 0.75 μL of IDV R primer (10 μM), 0.625 μL of IDV probe (10 μM), and 5.375 μL of nuclease-free water, was prepared. All real-time RT-PCR amplifications were performed using a StepOnePlus Real-Time PCR machine (Applied Biosystems, No: 4376600). The cycling conditions were at 95 °C for 5 min, followed by 45 cycles of 95 °C for 15 s and 60 °C for 1 min. Nuclease-free water was used as a negative control for all PCR runs without a template. Positive controls were obtained from samples submitted previously to the Department of Virology, Veterinary Faculty of Istanbul [8].

### 2.4. Sequencing and Phylogenetic Analysis

#### 2.4.1. Partial HEF Gene Sequencing

The HEF gene of IDV was targeted for sequence analysis. HEF-F and HEF-R primers reported by Zhai and others (2017) [25] were used for partial HEF gene sequencing (Table 2), with the expectation of obtaining a 496 bp product. Primers were positioned in the IDV PB1 gene using SnapGene (Version 7.2, IUC, Department of Virology) (Table 2). For this, a total volume of 25 μL of PCR mixture consisting of 12.5 μL of Platinum Hot Start PCR Master Mix (2X) (Invitrogen, Cat. No: 13000012), 5 μL of cDNA, 0.5 μL of forward primer (10 μM), 0.5 μL of reverse primer (10 μM), 5 μL of GC enhancer, and 1.5 μL of nuclease-free water was prepared. All PCR amplifications were performed in an Applied Biosystems™ SimpliAmp Thermal Cycler (Applied Biosystems, Cat. No: A24811). The cycling conditions were as follows: 95 °C for 5 min, followed by 35 cycles of 95 °C for 30 s for denaturation, 55 °C for 30 s for annealing, and 72 °C for 30 s for extension. Nuclease-free water was used as a negative control for all PCR runs without a template. Positive controls were obtained from samples previously submitted to the Department of Virology, Veterinary Faculty of Istanbul.

All products were analysed by 1.5% horizontal gel electrophoresis. The iBrightTM CL1500 Imaging System (Invitrogen, Cat. No: A44114) was used to visualise the PCR products in the gel. If 496 bp PCR bands were observed, the products were sent to a commercial company (MedSanTek, Istanbul, Türkiye) for sequencing.

#### 2.4.2. Whole HEF Gene Sequencing of IDV

The samples used for partial HEF gene (496 bp) sequencing were also analysed for whole HEF gene sequencing. For this study specifically, primers were designed and a method was established (Table 2). All products were analysed by 1.5% horizontal gel electrophoresis. The iBrightTM CL1500 Imaging System (Invitrogen, Cat. No: A44114) was used to visualise the PCR products in the gel. PCR products (618, 498, and 623 bp) were sent to a commercial company (MedSanTek) for sequencing. All HEF gene sequences were entered into GenBank and aligned using the MegAlign Pro (Version 17.6.1, DNAStar) and SnapGene (Version 7.2, IUC, Department of Virology) programmes, and a consensus sequence was determined. According to the consensus sequence, three new degenerate primer pairs for the 3 HEF gene regions, different than those described by Zhai and others (25), were designed. When all sequences obtained were assembled to align the whole HEF gene, primers 1 and 2 covered the region between 9 and 626 bp, the primers described by Zhai and others (25) covered the region between 582 and 1077 bp, primers 5 and 6 covered the region between 961 and 1458 bp, and primers 7 and 8 covered the region between 1366 and 1988 bp. Primer sets and their position on the aligned sequences are described in Table 4. The PCR conditions were the same as those used for partial HEF gene sequencing of IDV, as described in Section 2.4.1.

#### 2.4.3. Phylogenetic Analyses

To generate phylogenetic trees and compare the genotypic relationship between the IDV strains found in this study and IDV strains detected in other countries, the low-quality parts at the ends of the reverse and forward Sanger sequences of the HEF genes were cut off, and the forward and reverse sequences were combined using the Chromas Pro (Technelysium Version 2.1.10.1) programme. To perform genogrouping using Nucleotide Blast and GenBank, positive sample sequences were collected in Fasta form. All HEF gene sequences were submitted to GenBank and aligned using MegAlign Pro 17.6.1 (DNAStar) and SnapGene V7.2. Phylogenetic trees were generated using Maximum Likelihood, the RAxML method, and Bootstrap 1000 replication with MegAlign Pro (DNASTAR, Version 17.6). For the reference genes used in the phylogenetic trees, the methods reported by Ducatez et al. (2015) [5], Yılmaz et al. (2020) [8], and Yu et al. (2022) [18] were used. Partial HEF genes from 5 samples and whole HEF genes from 4 samples, for a total of 5 samples obtained in this study, were submitted to GenBank (for the 5 partial HEF genes of IDV, the Genbank Accession Numbers were PP574423.1, PP574424.1, PP832904.1, PP825810.1, PP825809.1; for the 4 whole HEF genes of IDV, the Genbank Accession Numbers were PQ766606.1, PQ766605.1, PQ766604.1, PQ766603.1).

### 2.5. Virus Isolation

#### 2.5.1. Virus Isolation Using HRT-18

Five nasal swabs found to be positive for IDV-RNA by real-time RT-PCR were used for to isolate the virus. HRT-18 cells were obtained from the National Veterinary School of Toulouse (ENVT, Toulouse, France). Cells were passaged on DMEM F-12 supplemented with 5% Foetal Bovine Serum (Gibco, Cat. No: A3160501) and 1% antibiotic solution (Gibco, Cat. No: 15140122). A total of four passages were performed on the cells from the swabs over a seven-day period. An inoculum was prepared as described previously [26]. For inoculation, the supernatant in the 24-well plate was removed, and the cells were washed with 500 μL of DPBS. After washing, 200 μL of inoculum/virus was added to the wells. The plates were incubated for 1 h in an incubator set at 37 °C and 5% CO_2_. After incubation, the virus on the cells was removed, and 1 mL of infection medium was gently mixed with the cells. The plates were incubated for 7 days in an incubator set at 37 °C and 5% CO_2_. The cells were examined daily under an inverted light microscope to determine their viability and the cytopathic effect. At the end of each passage, the cell culture was analysed by real-time RT-PCR for the presence of IDV-RNA and decreased Ct values.

#### 2.5.2. Virus Isolation in Embryonated Eggs

Conventional methods were used for egg inoculation. Inocula were prepared in the final passage supernatants of the IDV-inoculated HRT cells that were identified as positive by real-time RT-PCR. Commercial embryonated chicken eggs at the 10th day of development were conditioned in an egg incubator with 45–50% humidity at 37 °C. The inoculation area was disinfected with 70% ethanol solution, and 100 μL of the prepared dilutions were inoculated into the allantoic cavity of the 10-day embryonated eggs. Three eggs were used for each sample. Two of the eggs were stored at 33 °C, and one egg was stored at 37 °C in 45–50% humidity. The eggs were stored at 4 °C at the end of the 13th day, and the next day, allantoic fluids were collected by opening the eggs from the area in which the air sacs were located. The collected fluids were centrifuged at 3000 rpm for 20 min at 4 °C. Supernatants were passed through 0.45 μm filters and stored at −80 °C until required. The collected fluids were analysed by real-time RT-PCR for the presence of IDV-RNA and decreased Ct values.

### 2.6. ELISA

An optimised competitive ELISA was used to detect IDV antibodies in naturally infected animals using IDV-specific anti-IDV monoclonal antibodies (MAbs) supplied by the Istituto Zooprofilattico Sperimentale della Lombardia e dell’Emilia Romagna, Brescia, Italy, following the method reported by Moreno and others (2019) [6]. First, 50 μL of partially purified inactivated IDV (C172-LVS at a 1:80 dilution) antigen was coated onto plates and incubated overnight at 4 °C. The plates were washed with 200 μL of washing buffer (PBS supplemented with 0.05% (*v*/*v*) Tween 20) 3 times. The sera were tested at two dilutions: 1:10 and 1:20 (50 μL/well). Positive and negative control sera were added to each plate at the same dilutions. For the 100% control wells, 100 μL of buffer solution containing 1% yeast extract was used. The plates were covered and incubated for 60 min at 37 °C. After incubation, they were washed with 200 μL washing buffer 3 times. 50 μL HRPO-conjugate (2E12 HRP, 1:800 dilution) was added to the wells, and the plates were covered and incubated for 60 min at 37 °C. After incubation, the plates were washed with 200 μL of washing buffer 4 times. Then, 50 μL of substrate/chromogen solution (Sigma-Aldrich, Cat. No: P9187) was added to the wells, and the plates were covered and incubated for 60 min at room temperature. After 10 min, a stop solution was added to the wells, and the plates were read at a 490 nm wavelength using a microplate reader (SLT Spectra, Cat. No: 02523).

The test results were evaluated according to the following formula: % inhibition = 100 − (serum OD490/100% control OD490) × 100. After reading the spectrophotometer, an OD value ≥ 1 in 100% of the control wells was considered validation. Test sera with an inhibition value ≥ 65% at the 1:10 dilution were considered positive. The subsequent 1:20 dilution was used for the antibody level.

### 2.7. Hemagglutination and Hemagglutination Inhibition Assays

The hemagglutination assay was performed using the method established by Moreno and others [6]. Briefly, 50 μL of virus (2202-15) was used, and an RBC solution was prepared using chicken red blood cells. The hemagglutination titre was the dilution at which the RBCs were completely agglutinated. This titre was recorded as HA/50 μL of dilution. For the HI assay, sera were pre-treated to remove non-specific inhibitors using a receptor-destroying enzyme (RDE, Seiken, Cat. No: 370013). Then, 50 μL of serum was added to 200 μL of RDE and incubated for 18–20 h at 37 °C. After incubation, 150 μL of a 2.5% sodium citrate solution was added. The samples were heat-inactivated at 56 °C for 30 min and then serially diluted into the 96-well plates. Next, 25 μL of virus (2202-15) dilution, diluted as 4HA/25 μL, was added to all wells and incubated for 30 min. After incubation, 25 μL of erythrocyte solution was added to all wells and incubated for 30 min. HI was evaluated as described by Moreno and others [6]. An HI titer of >1/20 was considered positive.

### 2.8. Statistical Analyses

Statistical analyses were performed using GraphPad Prism software (Version 11, IUC, Department of Virology). Fisher’s exact test was used to compare demographic data and the IDV seropositivity of cattle and the symptoms observed in IDV-seropositive and -seronegative cattle. The independent relationship between the serology result, year, and age was further investigated using logistic regression analysis. In this study, two serological methods were used to detect antibodies to IDV in cattle sera.

## 3. Results

### 3.1. Clinical Findings

The animals examined in this study exhibited one or more of the following symptoms: dyspnoea, nasal discharge, cough, fatigue, and fever (Table 3). Amongst these symptoms, dyspnoea, nasal discharge, cough, and fatigue were observed in most of the animals.

### 3.2. Efficiency of RNA Extraction

To determine the matrix effect of the samples, RNA concentrations were measured after RNA extraction in six randomly selected samples. The A260/A280 RNA purity ratio was between 1.51 and 2.27 in the swab samples. In all samples with a purity ratio lower than 2.0 and below 100 ng/μL, the beta actin gene (housekeeping gene) was analysed by SYBR-Green real-time RT-PCR before or in parallel with IDV-RNA PCR to control the extraction efficiency, as described previously [27].

### 3.3. Detection of IDV-RNA

Among 279 nasal swabs, a total of 5 samples were found to be positive for IDV-RNA by real-time RT-PCR. All of the IDV-RNA-positive samples were collected from farms in Kırklareli. These samples were obtained from three different farms: one sample from 2020 from a small, family-owned farm, two samples from 2021 from an integrated farm, and two samples from 2024 from an integrated farm. The sample obtained from the small, family-owned farm in 2020 was from a <6-month-old animal that showed symptoms of respiratory distress, nasal discharge, cough, and fatigue, with a Ct value of 33.4. The samples taken from the integrated farm in 2021 were also from <6-month-old animals used for dairy who showed symptoms of respiratory distress, nasal discharge, cough, and fatigue, with Ct values of 25.3 and 34.3. The samples taken from the integrated farm in 2024 were from 6- to 12-month-old animals used for veal who showed dyspnoea, nasal discharge, coughing, fatigue, and fever, with Ct values of 28.6 and 33.8.

### 3.4. Virus Isolation

**Figure 1:**
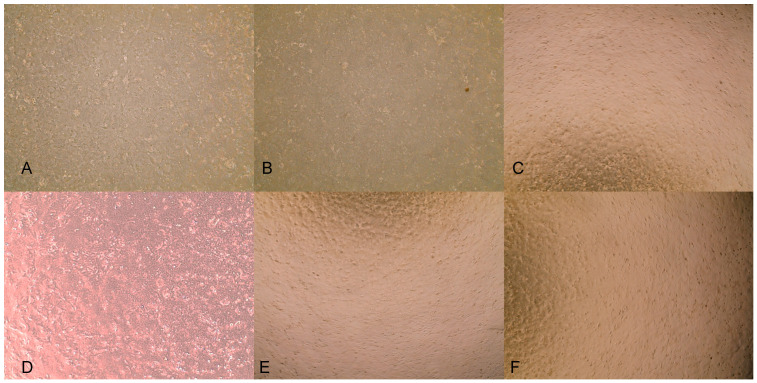
Images of virus-isolated samples in cell culture under a light microscope at 4X objective, demonstrating a lack of CPE (**A**) 403-10, (**B**) 2202-15, (**C**) 5950-1, (**D**) 5882-2, (**E**) 2202-4, (**F**) cell control.

At the virus isolation stage, cell cultures from swab samples stored in medium with codes 2202-15, 5950-1, 403-10, 5882-1, and 2202-4 had Ct values of 25.3, 28.6, 33.4, 33.8, and 34.3, respectively, according to real-time RT-PCR. When RT-PCR was used to check for the presence of IDV in the cell cultures at the end of the passages, all samples except for 2202-4 had a lower Ct value after each new passage compared to the previous one. A cytopathic effect (CPE) was not observed in any samples analysed or in negative control wells in these passages (Figure 1). Ultimately, four viruses (403-10, 2202-15, 5950-1, and 5882-2) were isolated from five samples.

We aimed to determine whether IDV present in samples that did not grow in cell culture could instead be propagated in embryonated eggs. Real-time RT-PCR results revealed that IDV from four samples grown in cell culture could also be grown in embryonated chicken eggs. Virus taken from sample 2202-4, which did not grow in cell culture after the last passage, grew when inoculated into 10-day-old embryonated chicken eggs. This result was confirmed with real-time RT-PCR, showing that the viruses can be grown using either method with equally good results.

### 3.5. Sequencing and Phylogenetic Analysis

#### 3.5.1. Partial HEF Gene Sequencing

Following virus isolation, for the purpose of partial sequencing of the HEF gene, the isolated 2202-4 virus was collected from the allantoic fluid, and other viruses were collected from cell fluids after the fourth passage. RT-PCR revealed a band at the 496 bp product was observed in samples 403-10, 2202-4, 2202-15, 5950-1, and 5882-2.

#### 3.5.2. Partial HEF Gene Phylogenetic Analysis

**Figure 2:**
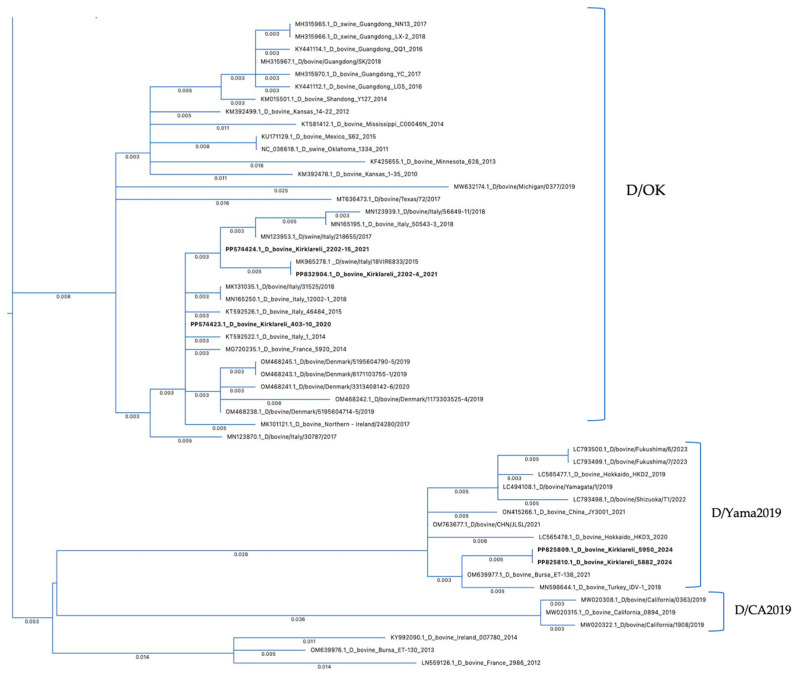
Phylogenetic tree based on partial HEF gene sequencing. Bold text indicates IDVs detected in this study. The Maximum Likelihood:RAxML method was used to construct the phylogenetic tree with 1000 Bootstrap replicates using MegAlign Pro Software (DNASTAR), using the HEF gene sequences of the strains detected in this study (PP574424.1, PP832904.1, PP574423.1, PP825809.1, and PP825810.1) and representative IDV strains reported elsewhere. The accession numbers shown in bold represent the IDVs detected in this study.

According to the sequencing data obtained, the sample coded 403-10 (GenBank Accession Number: PP574423.1), which tested positive, showed the highest identity, 99.73%, with IDV sequences reported in Italy (GenBank Accession Number: KT592522.1, KT592526.1, MK131035.1), France (GenBank Accession Number: MG720235.1), and Denmark (GenBank Accession Number: OM468238.1). The sample coded 2202-15 (GenBank Accession Number: PP574424.1) revealed the highest identity, 99.73%, with the virus detected in Italy (GenBank Accession Number: MN123953.1). The sample coded 2202-4 (GenBank Accession Number: PP832904.1) was identical to an Italian IDV strain (GenBank Accession Number: MK965278.1). Samples no. 5950-1 and no. 5882-2 (GenBank accession numbers PP825810.1 and PP825809.1, respectively) showed the highest nucleotide identity to IDV previously detected in Türkiye (GenBank accession numbers MN598644.1 and OM639977.1), with a 99.47–99.20% sequence similarity. Notably, sequence MN598644.1 had been submitted by our group earlier (Figure 2).

#### 3.5.3. Whole HEF Gene Sequencing

Whole HEF gene sequencing of the four IDVs detected in this study produced three bands at 618 bp, 498 bp, and 623 bp in samples 403-10, 2202-15, 5950-1, and 5882-2, respectively.

#### 3.5.4. Whole HEF Gene Phylogenetic Analysis

**Figure 3:**
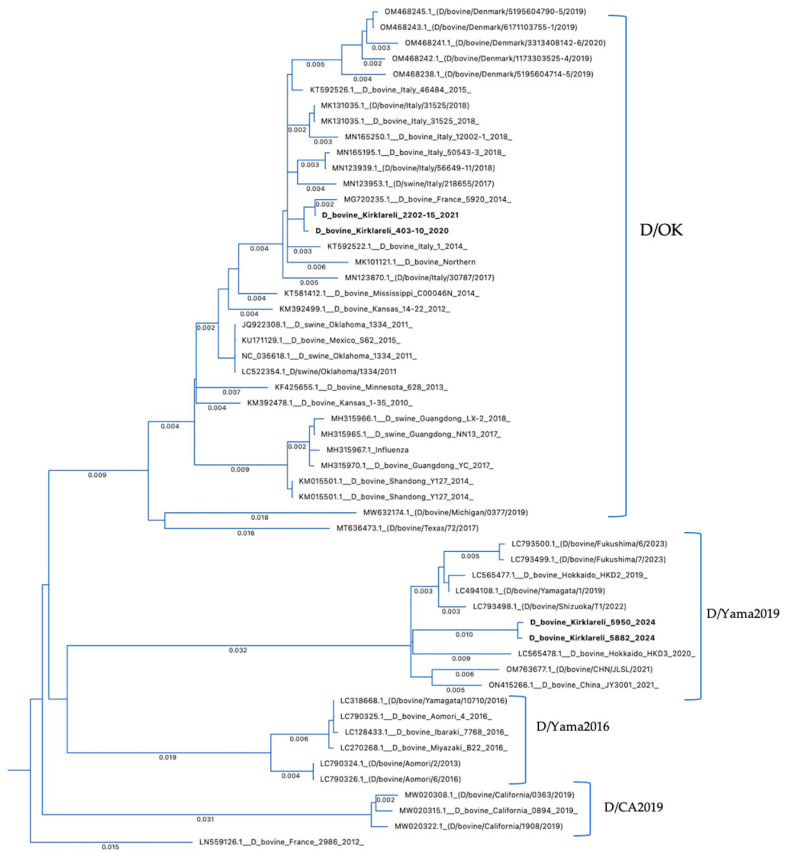
Phylogenetic tree based on whole HEF gene sequences. Bold text indicates the IDVs detected in this study. The Maximum Likelihood:RAxML method was used to construct the phylogenetic tree with 1000 Bootstrap replicates using MegAlign Pro Software (DNASTAR), using the whole HEF gene sequences of the strains detected in this study (PQ766606.1, PQ766605.1, PQ766604.1, and PQ766603.1) and representative IDV strains reported elsewhere. The accession numbers shown in bold represent the IDVs detected in this study.

The phylogenetic tree of the whole HEF gene was created as described above, using the Sanger sequencing results (Figure 3). Only the 498 bp band was displayed for the 2202-4 sample. According to these data, the whole HEF gene sequences of the samples coded 5950-1 and 5882-2 showed the closest identity, 98.58% with IDV sequences reported in Japan (GenBank Accession Number: LC494108) and the lowest identity, 92.72%, with IDV sequences reported in Italy (GenBank Accession Number: MN165250.1). The whole HEF gene sequences of sample 2202-15 showed the closest identity of 99.47% with an Italian strain (GenBank Accession Number: MK131035.1) and the lowest identity of 94.14% with Japanese IDV (GenBank Accession Number: LC565478.1) sequences. It was determined that the whole HEF gene sequence of sample 403-10 showed the closest identity, 99.53%, with an Italian strain (GenBank Accession Number: MK131035.1) and the lowest identity, 94.20%, with the Japanese IDV (GenBank Accession Number: LC565478.1) sequences.

#### 3.5.5. Amino Acid Comparison of the Whole HEF Gene

When the amino acid sequences of the HEF genes were compared using MegAlign Pro software (DNASTAR, version 17.6), only one amino acid difference was observed between samples no. 403-10 and no. 2202-15, collected in 2020 and 2021, respectively, across the 655-amino-acid HEF protein. In contrast, samples no. 5950-1 and no. 5882-2, collected in 2024, differed from the sample no. 403-10 by 25 amino acids and from sample no. 2202-15 by 24 amino acids (Supplementary Material, Figure S1). Phylogenetic analysis classified the samples no. 403-10 and no. 2202-15 within the D/OK lineage, whereas samples no. 5950-1 and no. 5882-2 clustered within the D/Yama2019 lineage. These findings indicate that the two D/OK lineage viruses are highly similar to each other, differing by only one amino acid, while they were clearly distinct from the D/Yama2019 lineage viruses, with 24–25 amino acid differences in the HEF protein.

### 3.6. ELISA

Amongst the 279 samples analysed by ELISA, antibodies to IDV were detected in 127 animals (45.5%) from 16 farms. It was observed that 22 of the positive blood sera belonged to animals from small family farms and 105 belonged to animals raised in integrated cattle farms. The ELISA revealed that, of the five samples (403-10, 2202-4, 2202-15, 5950-1, 5882-1) identified as positive by real-time RT-PCR, four of the serum samples, 403-10, 2202-4, 5950-1, and 5882-2, had inhibition levels of 69.44%, 89.3%, 69.03, and 64.45%, respectively. The other sample (2202-15) had an inhibition level of %53.67, below the positive cut-off value of 65%.

### 3.7. Hemagglutination Inhibition

As a result of the hemagglutination test, the hemagglutination titre of the virus produced from the 2202-15 sample was determined to be 256 HAU/50 μL, and the virus was diluted to 4HAU/25 μL for use in the HI test. The virus was diluted 1/32 and added to PBS. An amount of virus sufficient for 154 samples was diluted, and HI titres ranging from 2 to 9 were observed in the collected sera. After RDE treatment, the titres of 154 sera were >1/20 (n = 154/279), and the positivity rate was 55.19%.

### 3.8. Statistical Analysis

Fisher’s exact test analysis identified the study year (*p* = 0.0008), geographical location (*p* < 0.0001), and farm type (*p* < 0.0001) as statistically significant variables associated with IDV seropositivity, as determined by ELISA. An increase in seroprevalence from 48% in both 2020 and 2021 to 90% in 2024 was observed. At the provincial level, seroprevalence was the highest in Kırklareli (60%) and the lowest in Tekirdağ (18%). Seroprevalence on integrated farms (62%) was approximately twice that on small, family-owned farms (31%). Sex, age group, and animal category were not significantly associated with seropositivity (*p* > 0.9999, *p* = 0.1961, and *p* > 0.9999, respectively) (Table 4).

Regarding clinical findings, nasal discharge (*p* = 0.0356) and cough (*p* = 0.0339) were the only symptoms that differed significantly between seropositive and seronegative animals, whereas dyspnoea, weakness, and fever showed no significant difference (*p* = 0.1194, *p* = 0.0692, and *p* = 0.8450, respectively). Multivariable logistic regression analysis revealed that animals sampled in 2024 had more than eightfold higher odds of seropositivity compared with the 2020 reference year (OR = 8.308; 95% CI: 1.860–59.53; *p* = 0.0042), while no significant difference was observed for 2021 (OR = 0.847; 95% CI: 0.367–1.944; *p* = 0.6941). Age group was not identified as an independent predictor of seropositivity (Table 5 and Table 6).

Fisher’s exact test of the HI test results identified the study year (*p* = 0.0012), geographical location (*p* < 0.0001), and farm type (*p* < 0.0001) as statistically significant variables associated with IDV seropositivity by HI. Seroprevalence increased from 40% in 2020 (10/25; 95% CI: 21–59%) and 54% in 2021 (126/234; 95% CI: 47–60%) to 90% in 2024 (18/20; 95% CI: 77–103%). Regional variation was pronounced, with seroprevalence ranging from 18% in Tekirdağ (3/17; 95% CI: 0–36%) and 33% in Edirne (14/42; 95% CI: 19–48%) to 55% in İstanbul (11/20; 95% CI: 33–77%) and 63% in Kırklareli (126/200; 95% CI: 56–70%). Cattle on integrated farms exhibited a seroprevalence of 67% (124/185; 95% CI: 60–74%), approximately twice that found in small farms (32%, 30/94; 95% CI: 22–41%). Sex (*p* = 0.0778), age group (*p* = 0.2918), and animal category (*p* = 0.0778) were not significantly associated with HI seropositivity (Table 7).

Regarding the association between HI serostatus and clinical signs, cough was the only sign that differed significantly between HI-positive and HI-negative animals (85% vs. 94%; *p* = 0.0338), while dyspnoea, nasal discharge, weakness, and fever had no significant difference (*p* = 0.4314, *p* = 0.1003, *p* = 0.1703, and *p* = 0.5547, respectively). Multivariable logistic regression analysis confirmed the sampling year as the sole independent predictor of HI seropositivity, with animals sampled in 2024 showing a more than thirteenfold increase in the odds of seropositivity relative to the 2020 reference year (OR = 13.50; 95% CI: 3.021–97.53; *p* = 0.0003). In contrast, no significant difference was determined for 2021 (OR = 1.750; 95% CI: 0.763–HI; *p* = 0.1871). Age group was not identified as an independent predictor in either age category examined (3–6 years: OR = 0.601, *p* = 0.1390; 6–12 years: OR = 0.540, *p* = 0.1502) (Table 8 and Table 9).

## 4. Discussion

Influenza D virus was first isolated in 2011 and has since been detected in different species. Cattle are the main host, and IDV is one of the factors in the occurrence of BRDC, which causes major economic losses in the cattle industry worldwide [1,28,29]. BRDC, which is caused by viruses such as BoHV-1, BVDV, BRSV, BCoV, and BIPV3 in addition to IDV, is tested in cattle samples in serological and molecular surveillance studies in cattle worldwide. For this purpose, ELISA and PCR tests are mostly used [15].

IDV has been investigated frequently in recent years, especially in America, Europe, and China, but a limited number of studies have been conducted in Türkiye [8,17]. The studies performed in Türkiye have not examined the immune response to IDV; therefore, this study was conducted to investigate the prevalence, diagnosis, isolation, molecular characterisation, and immune response to IDV in cattle in farms located in the Marmara Region, along the European border of Türkiye.

Molecular tests are frequently used in IDV diagnosis and molecular epidemiological studies worldwide. Real-time RT-PCR and RT-PCR are the most commonly used molecular tests [15,24,30]. Different results have been obtained in molecular epidemiology studies conducted in different countries using PCR. In the United States, a positivity rate of 4.8% was reported in 208 lung, nasal, and oropharyngeal swab samples collected from 12 different states in 2014 [31]. In Canada, the positivity rate among 883 nasal and oropharyngeal swabs collected between 2017 and 2020 was 5.3% [32]. A study in Mexico reported positivity rates of 29.6% and 3.8% in nasal swabs from 27 animals with respiratory symptoms and nasal swabs from 26 asymptomatic animals, respectively [9]. In South America, IDV was first detected in one out of nine nasal swabs obtained on a Brazilian cattle farm experiencing a BoHV-1 outbreak in 2022 [33]. In Europe, IDV was first detected in France in 134 lung and nasal swabs and tracheal aspirate fluid samples collected between 2010 and 2013, with a 4.5% positivity rate [5]. In a study conducted in Italy between 2018 and 2019 with 664 nasal swabs, 250 lung tissue samples, and 22 bronchoalveolar fluid samples, positivity rates of 13% and 28% were detected in nasal swabs and lung tissues, respectively [34]. In a study performed in Denmark in 2022, a positivity rate of 12% was found in 100 nasal swab samples [35]. In Ireland in 2018, a positivity rate of 5.6% was reported following the analyses of 320 nasal swab samples [36]. In a study conducted by Studer et al. in Switzerland in 2021, the positivity rate was 4.1% among 764 nasopharyngeal swab samples % [37]. In the UK, a prevalence of 8.7% was reported in a sample pool consisting of 104 nasal swabs, as well as trachea and lung samples [35]. In a recent study conducted in Spain in 316 samples, including nasal swabs and bronchoalveolar lavage and lung tissue samples, a positivity rate of 12% was reported [19]. In a study performed in China in 2014, IDV was detected in 10.4% of nasal swab samples taken from 230 animals showing respiratory symptoms [25]. In a study conducted by Lim et al. (2023) [38], a positivity rate of 1.4% was reported in nasal swabs out of 999 samples consisting of nasal swabs and lung tissues [35]. In Africa, IDV was first detected in Nigeria in 2023, determining a positivity rate of 35.2% among 80 cattle [39]. IDV was first detected in Türkiye in 2020 by Yılmaz and others (2020) [8], and the positivity rate was reported as 4% in a study covering 76 samples [8]. In another study conducted in Türkiye, a positivity rate of 0.9% was reported among 219 nasal swab samples collected between 2012 and 2021 [17]. In this present study, RT-PCR analyses were performed on 279 nasal swab samples taken from cattle showing respiratory symptoms such as dyspnoea, nasal discharge, cough, fatigue, and fever, and a positivity rate of 1.79% (*n* = 5/279) for IDV-RNA. When these results are compared with previously published results, the positivity rate is lower than that found in the 2020 study by Yılmaz and others (2020) [8] but higher than in the results of the study conducted by Yeşilbağ and others (2022) [17]. The reason for this may be that the number of samples collected in the study conducted by Yılmaz et al. (2020) [8] was 76, and Yeşilbağ and others (2022) [17] investigated 219 archival samples, meaning that the viral RNA may have degraded during storage. It is also important to note that low virus positivity according to qRT-PCR results does not mean that the circulation of the pathogen is low; it is very dependent upon the timing of sampling, as the virus shedding period is short (2 weeks under experimental conditions [14]). There was also bias preventing proposer comparison between the referenced studies due to the samples selected: symptomatic and asymptomatic animals are expected to have different virus positivity rates.

In this present study, the results of the sequencing performed for phylogenetic analysis revealed that three samples collected in 2020 and 2021 clustered in the D/OK genogroup, and two samples collected in 2024 were in the D/Yama2019 genogroup. These results indicate that D/OK and D/Yama2019 strains are circulating in Türkiye. The samples clustered in the D/OK genogroup showed 99.73–100% identity with influenza D viruses reported in Italy [40,41], France [14], and Denmark [42]. Additionally, samples clustered in the D/Yama2019 genogroup showed 95.48–99.7% identity with influenza D viruses reported in Türkiye [8,17]. More samples were analysed in this study compared to previous molecular studies performed in Türkiye, and while a lower positivity rate was detected compared to the study conducted in 2020 [8], a higher positivity rate was detected compared to the study conducted in 2022 [17]. With the phylogenetic analyses performed in this study, the whole HEF gene was also sequenced. These results indicated 99.47–99.53% identity with IDV reported in Italy [43] from positive samples from 2020 and 2021 and 98.58% identity with IDV reported in Japan from positive samples from 2024 [44]. While D/Yama2019 strains were detected in previous studies performed in Türkiye, data from this study show that the D/OK strain was also circulating between 2020 and 2021. The detection of D/Yama2019 strain in samples taken in 2024 indicates that both strains are currently circulating in Türkiye. Both strains may be circulating due to the import of live cattle and cattle products. In addition, the IDV numbered PP832904.1 detected in our study was found to be identical to MK965278.1, which was of pig origin and detected in Italy [40]. The origin of the IDV strains circulating in Türkiye requires further investigation.

HRT-18, ST, Vero, MDCK, and MDBK cells are usually used to isolate IDV in cell cultures [1,13,45]. In IDV isolation studies performed in different countries with HRT-18 and ST cells, CPE was not observed in HRT-18 cells but was observed in ST cells, and real-time RT-PCR, RT-PCR, and Hemagglutination tests were used to confirm viral growth [7,13,31,45]. Several virus isolation studies were also conducted using MDCK, MDBK, and Vero cells. In a study performed by Jiang and others [46], CPE was observed in Vero and MDBK cells but not in MDCK cells. These outcomes draw attention to the fact that virus isolation can fail using samples with real-time RT-PCR results of 29–36 Ct despite low Ct results in HRT-18 cell cultures [5,47]. In this study, this issue was considered, and Ct values were monitored until the end of the fourth passage, with low Ct values (11–30) being detected. In addition, isolation studies were carried out in embryonated eggs using the same samples. Virus shedding in the upper respiratory tract of infected calves occurs over a period of 4–6 days. Therefore, the sampling time is another important factor in virus isolation [7,47].

In this, embryonated chicken eggs were used for IDV isolation, as in other studies [3,48]. Inocula were prepared from five nasal swab samples that were found to be positive by real-time RT-PCR. Cell culture supernatants of the samples were inoculated into embryonated chicken eggs after the fourth passage. It was not possible to isolate the virus from one sample in cell culture; however, it could be isolated when inoculated into embryonated chicken eggs, showing that there was virus in the cell culture supernatant, but its titre was below the threshold needed for real-time RT-PCR detection. In this case, isolation of this virus in cell culture might have been possible if more passages were performed. The data obtained in this study show that viruses can be isolated using both methods, without indicating if one is more effective.

Many studies have reported different seroprevalence rates in different countries [15,49]. In a study conducted in the United States in 2014 and 2015, a seroprevalence of 77.5% was determined in 1992 cattle sera collected from 41 states [50]. In Argentina, the seroprevalence was 68% among 165 cattle [51]. In Europe, IDV was first reported in France in 2014, and various studies were subsequently conducted in Italy, Denmark, Luxembourg, Ireland, Sweden, Switzerland, and the United Kingdom [15]. In Europe, the highest IDV seroprevalences were in Ireland, Italy, and Luxembourg, at 94.6%, 92.4%, and 82.5%, respectively [38,52,53]. In France, 47.2% seropositivity was found in 3000 cattle analysed [54]. In Sweden, 461 milk samples in 2019 and 338 milk samples in 2020 were analysed, and 32% and 40% were seropositive, respectively [55]. In a recent study conducted in Poland, 45.2% of 755 serum samples were seropositive [56]. In Asia, a seroprevalence of 5.9% among 193 serum samples was reported in China (2014) [24]. In a serological study conducted in 1927 in Japan, serum samples collected between 2010 and 2016 revealed IDV seropositivity of 30.5% [57]. In Korea, seroprevalence was 54.7% in 742 serum samples [38]. In Africa, the highest seroprevalence was reported in Morocco, where it was 35% in a study conducted in 200 sera, followed by Togo, where 10.4% of 201 serum samples were positive, and Benin, where 1.9% of 201 serum samples were positive [58].

IDV seroprevalence has not been studied in Türkiye before, and there is currently no commercial serological test that measures antibody response in cattle. We therefore used *in-house* ELISA and HI tests to detect antibodies to IDV in this study; these tests have been used by others [5]. In this study, the ELISA and HI assays were used to detect IDV antibodies in cattle sera. The ELISA found a seropositivity rate of 45.5% (*n* = 127/279) in 279 cattle sera. Titre determination was performed with the HI test in all serum samples as well, with a result of >1/20 in 154 of 279 sera.

These data obtained by ELISA and HI are the first results showing the seroprevalence—in other words, immune response to natural IDV infection—in cattle in Türkiye. The results of the serological tests performed in this study provide data that will be useful for the establishment of a commercial ELISA kit system for IDV detection in the future. The results of these ELISA and HI assays were similar to the results of the study conducted in Korea, with lower values observed compared to results published in Europe and in the United States and higher seropositivity compared to China, Japan, Africa, and the Middle East. Importantly, considering the very limited 2024 cohort tested in this study (*n* = 20), the results should not be overinterpreted.

Statistical analysis of this study’s ELISA and HI results identified the study year, geographical location, and farm type as statistically significant variables associated with IDV seropositivity. At the provincial level, seroprevalence was the highest in Kırklareli (60%) and the lowest in Tekirdağ (18%), a geographical heterogeneity that may reflect regional differences in husbandry conditions, biosecurity practices, and animal movement intensity. Seroprevalence on integrated farms was approximately twice that recorded on small, family-owned farms, suggesting that the higher animal density and shared equipment use inherent to intensive production systems facilitate viral spread and transmission. It was also noted in other studies that countries in which intensive cattle farming is common show higher seroprevalence compared to regions that use traditional farming practices [54,56]. Once more, the fact that symptomatic animals were sampled in this study introduces bias when considering seroprevalence at a global level. Further studies on animals with and without clinical signs are warranted to allow for the assessment of real seroprevalence in Türkiye.

With respect to clinical findings, nasal discharge and cough were the only symptoms that differed significantly between seropositive and seronegative animals by ELISA, while cough was the only sign that differed significantly between HI-positive and HI-negative animals. A study exploring the pathogenesis of IDV reported that a strong and frequent cough is a predominant symptom [59]. Another study investigating the bull-to-pig transmission of IDV emphasised the importance of considering the occurrence of cough [60]. Multivariable logistic regression analysis revealed that the animals sampled in 2024 had more than eightfold higher odds of seropositivity compared with the 2020 reference year. It should be noted that an increasing positivity rate over the years is an important factor for consideration [61,62]. Taken together, these findings indicate that the ELISA- and HI-based results further substantiate the temporal escalation of IDV transmission, particularly within intensive production systems.

In conclusion, different lineages (D/OK, D/Yama2019) of IDV are currently circulating in the Thrace region of Türkiye. The ELISA and HI results show that about 50% of cattle are exposed to IDV. Overall, the results indicate that IDV is a threat to the health of cattle, and more studies on the contribution of IDV to BRDC in cattle are needed, in addition to the development of vaccines and diagnostic tests. Given the absence of influenza D virus (IDV) vaccines, these findings emphasise the importance of continued surveillance and diagnostic testing of cattle to limit viral spread, protect the health of cattle, and reduce economic losses associated with IDV.

## Figures and Tables

**Table 1:** Primers and probes used for the detection of IDV and position in PB1 gene.

Primers and Probe	Target Gene	Primer Design	Product Size	Positions *	Reference
IDV-F	PB1	5′-TGG ATG GAG AGT GCT GCT TC-3′	109 bp	1215–1234	[24]
IDV-R		5′-GCC AAT GCT TCC TCC CTG TA-3′		1304–1323	
IDV Probe		5′-FAM- CAT GTT AAA CAT TCC CAT CAG CAT TCC T−BHQ1-3′		1270–1243	

* As described in Faccini and others [24].

**Table 2:** Primers used for partial and whole HEF gene sequencing.

Primers	Primer Design	Product Size	Positions	Reference
HEF-F	5′-AAC CRC ATC TTC TTG TTC TTC A-3′	496 bp	582–1077	[25]
HEF-R	5′-TGC TTC TTC WGT GGC ATT ATC T-3′			
Consensus_Primer_1_F	5′-GCTTCTAGCAACAATYACAGC-3′	618 bp	9–29	This study
Consensus_Primer_2_R	5′-GGATTAACAGAAGATTCTGCATACC-3′		602–626	
Consensus_Primer_5_F	5′-TAYTGCTTCGACACTGATGG-3′	498 bp	961–980	
Consensus_Primer_6_R	5′-AYTTGATCTTCCCCAGAAGTAAC-3′		1436–1458	
Consensus_Primer_7_F	5′-ATCTTCGGAATAGATGACTTARTCTTC-3′	623 bp	1366–1392	
Consensus_Primer_8_R	5′-TTGCAACAGAYCCAAATCACAC-3′		1967–1988	

**Table 3:** Location, age, number of samples, and symptoms of the cattle analysed in this study.

Location	Age	Number of Samples	Clinical Symptoms
		(Nasal Swabs and Blood)	
İstanbul	6–12	13	Dyspnoea, nasal discharge, cough, fatigue
Kırklareli	3–6	12	Dyspnoea, nasal discharge, cough, fatigue
Tekirdağ	3–6	17	Dyspnoea, nasal discharge, cough, fatigue
Kırklareli	0–3	9	Nasal discharge, cough, fatigue
Kırklareli	3–6	40	Dyspnoea, nasal discharge, cough, fatigue
Kırklareli	3–6	18	Dyspnoea, nasal discharge, cough, fatigue
Edirne	0–3	15	Nasal discharge, cough, fatigue
Kırklareli	3–6	23	Dyspnoea, nasal discharge, cough, fatigue
Kırklareli	6–12	3	Dyspnoea, nasal discharge, cough, fatigue
Kırklareli	3–6	12	Dyspnoea, nasal discharge, cough, fatigue
Kırklareli	3–6	14	Dyspnoea, nasal discharge, cough, fatigue
Kırklareli	3–6	9	Dyspnoea, nasal discharge, cough, fatigue
Kırklareli	3–6	20	Dyspnoea, nasal discharge, coughing, fatigue
İstanbul	6–12	7	Nasal discharge, cough, fatigue, fever
Edirne	6–12	10	Nasal discharge, cough, fatigue, fever
Edirne	3–6	6	Nasal discharge, cough, fatigue
Edirne	3–6	11	Nasal discharge, cough, fatigue
Kırklareli	0–3	20	Nasal discharge, cough, fatigue
Kırklareli	0–12	20	Dyspnoea, nasal discharge, cough, fatigue, fever

**Table 4:** The estimated IDV seroprevalence of ELISA results according to year and cattle demographic variables (CI, Confidence Interval).

Variables	Level	N	Negative	Positive	% Positive	95%CI		Fisher’s Exact Test	Statistically Significant
						Lower	Upper	*p*-Value	(*p* < 0.05)
Study year	2020	25	12	12	48	28	68	0.0008	Yes
	2021	234	122	112	48	41	54		
	2024	20	2	18	90	77	103		
Gender	Female	129	53	76	59	50	67	>0.9999	No
	Male	81	33	48	59	49	70		
Location	Edirne	42	31	11	26	13	39	<0.0001	Yes
	Istanbul	20	10	10	50	28	72		
	Kırklareli	200	81	119	60	53	66		
	Tekirdağ	17	14	3	18	0	36		
Age (months)	0–3	44	16	28	64	49	78	0.1961	No
	3–6	188	97	91	48	41	56		
	6–12	47	23	24	51	37	65		
Farm type	Small, family-owned	94	65	29	31	22	40	<0.0001	Yes
	Integrated	185	71	114	62	55	69		
Dairy/Veal Calf	Dairy	129	53	76	59	50	67	>0.9999	No
	Veal Calf	81	33	48	59	49	70		

**Table 5:** The estimated prevalence of clinical signs according to cattle ELISA status.

Variables	ELISA Positive N	Number of Animals Affected	95%CI			ELISA Negative N	Number of Animals Affected	95%CI			Fisher’s Exact Test *p*-Value	Statistically Significant (*p* < 0.05)
			95%CI	Lower	Upper			95%CI	Lower	Upper		
Dyspnoea	143	106	74	73	76	136	89	65	64	67	0.1194	No
Nasal discharge	143	128	90	89	90	136	131	96	96	97	0.0356	Yes
Cough	143	122	85	84	86	136	127	93	93	94	0.0339	Yes
Fatigue	143	128	90	89	90	136	130	96	95	96	0.0692	No
Fever	143	14	10	9	11	136	15	11	10	12	0.845	No

**Table 6:** The estimated risk of IDV seropositivity by ELISA according to year and age.

Variables	Level	Odds Ratios	95%CI Lower	95%CI Upper	Logistic Regression *p*-Value	Statistically Significant (*p* < 0.05)
Study year	2020	1	-	-	-	-
	2021	0.8474	0.3666	1.944	0.6941	No
	2024	8.308	1.86	59.53	0.0042	Yes
Age (months)	0–3	1	-	-	-	
	3–6	0.5361	0.2674	1.045	0.0672	No
	6–12	0.5963	0.2545	1.373	0.225	No

**Table 7:** The estimated IDV seroprevalence of HI results according to year and cattle demographic variables.

Variables	Level	N	Negative	Positive	% Positive.	95%CI		Fisher’s Exact Test *p*-Value	Statistically Significant (*p* < 0.05)
						Lower	Upper		
Study year	2020	25	15	10	40	21	59	0.0012	Yes
	2021	234	108	126	54	47	60		
	2024	20	2	18	90	77	103		
Gender	Female	129	40	89	69	61	77	0.0778	No
	Male	81	35	46	57	46	68		
Location	Edirne	42	28	14	33	19	48	<0.0001	Yes
	Istanbul	20	9	11	55	33	77		
	Kırklareli	200	74	126	63	56	70		
	Tekirdağ	17	14	3	18	0	36		
Age (months)	0–3	44	15	29	66	52	80	0.2918	No
	3–6	188	87	101	54	47	61		
	6–12	47	23	24	51	37	65		
Farm type	Small family owned	94	64	30	32	22	41	<0.0001	Yes
	Integrated	185	61	124	67	60	74		
Dairy/Veal Calf	Dairy	129	40	89	69	61	77	0.0778	No
	Veal Calf	81	35	46	57	46	68		

**Table 8:** The estimated seroprevalence of clinical signs according to HI status in cattle.

Variables	HI Positive N	Number of Affected Animals	95%CI			HI Negative N	Number of Affected Animals	95%CI			Fisher’s Exact Test *p*-Value	Statistically Significant (*p* < 0.05)
			% 95CI	Lower	Upper			% 95CI	Lower	Upper		
Dyspnoea	154	111	72	70	74	125	84	67	65	69	0.4314	No
Nasal discharge	154	139	90	90	91	125	120	96	96	96	0.1003	No
Cough	154	131	85	84	86	125	117	94	93	94	0.0338	Yes
Fatigue	154	139	90	90	91	125	119	95	95	96	0.1703	No
Fever	154	18	12	11	13	125	11	9	8	10	0.5547	No

**Table 9:** The estimated risk of IDV seropositivity by HI according to year and age.

Variables	Level	Odds Ratios	95%CI Lower	95%CI Upper	Logistic Regression *p*-Value	Statistically Significant (*p* < 0.05)
Study year	2020	1	-	-	-	
	2021	1.75	0.763	HI	0.1871	No
	2024	13.5	3.021	97.53	0.0003	Yes
Age (months)	0–3	1	-	-	-	
	3–6	0.6005	0.296	1.177	0.139	No
	6–12	0.5397	0.2283	1.249	0.1502	No

## Data Availability

Data are available upon reasonable request from the corresponding authors.

## Supplementary Materials

The following supporting information can be downloaded at: https://www.mdpi.com/article/10.3390/v18060626/s1, Figure S1. Differences in amino acid sequences of whole HEF gene sequences.

## References

[1] Hause B.M., Collin E.A., Liu R., Huang B., Sheng Z., Lu W., Wang D., Nelson E.A., Li F. (2014). Characterization of a Novel Influenza Virus in Cattle and Swine: Proposal for a New Genus in the Orthomyxoviridae Family. mBio.

[2] Ferguson L., Olivier A.K., Genova S., Epperson W.B., Smith D.R., Schneider L., Barton K., McCuan K., Webby R.J., Wan X.F. (2016). Pathogenesis of Influenza D Virus in Cattle. J. Virol..

[3] Asha K., Kumar B. (2019). Emerging Influenza D Virus Threat: What We Know So Far!. J. Clin. Virol..

[4] Liu R., Sheng Z., Huang C., Wang D., Li F. (2020). Influenza D Virus. Curr. Opin. Virol..

[5] Ducatez M.F., Pelletier C., Meyer G. (2015). Influenza D Virus in Cattle, France, 2011–2014. Emerg. Infect. Dis..

[6] Moreno A., Lelli D., Lavazza A., Sozzi E., Zanni I., Chiapponi C., Foni E., Capucci L., Brocchi E. (2019). MAb-based Competitive ELISA for the Detection of Antibodies Against Influenza D Virus. Transbound. Emerg. Dis..

[7] Ferguson L., Eckard L., Epperson W.B., Long L.P., Smith D., Huston C., Genova S., Webby R., Wan X.F. (2015). Influenza D Virus Infection in Mississippi Beef Cattle. Virology.

[8] Yılmaz A., Umar S., Turan N., Aydın O., Tali H.E., Oğuzoğlu T.C., Yılmaz H., Richt J.A., Ducatez M.F. (2020). First Report of Influenza D Virus Infection in Turkish Cattle with Respiratory Disease. Res. Vet. Sci..

[9] Mitra N., Cernicchiaro N., Torres S., Li F., Hause B.M. (2016). Metagenomic Characterization of the Virome Associated with Bovine Respiratory Disease in Feedlot Cattle Identified Novel Viruses and Suggests an Etiologic Role for Influenza D Virus. J. Gen. Virol..

[10] Pardon B., De Bleecker K., Dewulf J., Callens J., Boyen F., Catry B., Deprez P. (2011). Prevalence of Respiratory Pathogens in Diseased, Non-vaccinated, Routinely Medicated Veal Calves. Vet. Rec..

[11] Kurcubic V., Djokovic R., Ilic Z., Vaskovic N., Petrovic M. (2019). Bovine Respiratory Disease Complex (BRDC): A Review of Lung Lesions and Reducing of Quality of Carcasses. Biotechnol. Anim. Husb..

[12] Umar S., Yuan R., Li Y., Gao D., Anderson B.D. (2023). No Influenza D Virus and Human Seasonal Influenza A Virus (H1N1, H3N2) Were Detected among Dogs, Kunshan, China. Acta Vet. Eurasia.

[13] Holwerda M., Kelly J., Laloli L., Stürmer I., Portmann J., Stalder H., Dijkman R. (2019). Determining the Replication Kinetics and Cellular Tropism of Influenza D Virus on Primary Well-differentiated Human Airway Epithelial Cells. Viruses.

[14] Salem E., Hägglund S., Cassard H., Corre T., Näslund K., Foret C., Gauthier D., Pinard A., Delverdier M., Meyer G. (2019). Pathogenesis, Host Innate Immune Response, and Aerosol Transmission of Influenza D Virus in Cattle. J. Virol..

[15] Kwasnik M., Rola J., Rozek W. (2023). Influenza D in Domestic and Wild Animals. Viruses.

[16] Huang C., Yu J., Hause B.M., Park J.Y., Sreenivasan C., Uprety T., Sheng Z., Wang D., Li F. (2021). Emergence of New Phylogenetic Lineage of Influenza D Virus with Broad Antigenicity in California, United States. Emerg. Microbes Infect..

[17] Yesilbag K., Toker E.B., Ates O. (2022). Recent Strains of Influenza D Virus Create a New Genetic Cluster for European Strains. Microb. Pathog..

[18] Yu J., Li T., Wen Z., Wu S., Wang Z., Zheng J., Chen M., Chen F., Wei W.K., Zhai S.L. (2022). Identification of D/Yama2019 Lineage-like Influenza D Virus in Chinese Cattle. Front. Vet. Sci..

[19] Benito A.A., Monteagudo L.V., Lázaro-Gaspar S., Garza-Moreno L., Antón-Baltanás N., Quílez J. (2026). First Detection and Genetic Characterization of Influenza D Virus in Cattle in Spain. Vet. Sci..

[20] Ishida H., Murakami S., Kamiki H., Matsugo H., Takenaka-Uema A., Horimoto T. (2020). Establishment of a Reverse Genetics System for Influenza D Virus. J. Virol..

[21] Turkish Statistical Institute (TÜİK) (2025). Hayvansal Üretim Istatistikleri [MEDAS Database]. https://biruni.tuik.gov.tr/medas/?kn=101&locale=tr.

[22] Oruc S., Yalcin E. (2021). Extreme Precipitation Indices Trend Assessment Over Thrace Region, Turkey. Acta Geophys..

[23] Eroğlu İ. (2024). Trend Analysis of Precipitation in the Thrace Peninsula. J. Geogr..

[24] Faccini S., De Mattia A., Chiapponi C., Barbieri I., Boniotti M.B., Rosignoli C., Franzini G., Moreno A., Foni E., Nigrelli A.D. (2017). Development and Evaluation of a New Real Time RT-PCR Assay for Detection of Proposed Influenza D Virus. J. Virol. Methods..

[25] Zhai S.L., Zhang H., Chen S.N., Zhou X., Lin T., Liu R., Lv D.H., Wen X.H., Wei W.K., Wang D. (2017). Influenza D Virus in Animal Species in Guangdong Province, Southern China. Emerg. Infect. Dis..

[26] Yilmaz S.G., Yilmaz A., Karadag G., Turan N., Richt J., Yilmaz H. (2025). Molecular Characterization of BCoV Infecting Vaccinated and Non-vaccinated Cattle in Thrace District Türkiye and Isolation of Field Strains. Virol. J..

[27] Aydin O., Yilmaz A., Turan N., Richt J.A., Yilmaz H. (2024). Molecular Characterisation and Antibody Response to Bovine Respiratory Syncytial Virus in Vaccinated and Infected Cattle in Turkey. Pathogens.

[28] Ishida H., Murakami S., Kamiki H., Matsugo H., Katayama M., Sekine W., Ohira K., Takenaka-Uema A., Horimoto T. (2021). Construction of an Influenza D Virus with an Eight-segmented Genome. Viruses.

[29] Katayama M., Murakami S., Ishida H., Matsugo H., Sekine W., Ohira K., Takenaka-Uema A., Horimoto T. (2024). Antigenic Commonality and Divergence of Hemagglutinin-Esterase-Fusion Protein Among Influenza D Virus Lineages Revealed Using Epitope Mapping. J. Virol..

[30] Yu J., Hika B., Liu R., Sheng Z., Hause B.M., Li F., Yu J., Li F., Wang D. (2020). The First Decade of Research Advances in Influenza D Virus. J. Gen. Virol..

[31] Collin E.A., Sheng Z., Lang Y., Ma W., Hause B.M., Li F. (2015). Cocirculation of Two Distinct Genetic and Antigenic Lineages of Proposed Influenza D Virus in Cattle. J. Virol..

[32] Saegerman C., Gaudino M., Savard C., Broes A., Ariel O., Meyer G., Ducatez M.F. (2022). Influenza D Virus in Respiratory Disease in Canadian Province of Québec, Cattle: Relative Importance and Evidence of New Reassortment Between Different Clades. Transbound. Emerg. Dis..

[33] da Silva M.S., Mosena A.C.S., Baumbach L., Demoliner M., Gularte J.S., Pavarini S.P., Driemeier D., Weber M.N., Spilki F.R., Canal C.W. (2022). Cattle Influenza D Virus in Brazil Is Divergent from Established Lineages. Arch. Virol..

[34] Chiapponi C., Faccini S., Fusaro A., Moreno A., Prosperi A., Merenda M., Baioni L., Gabbi V., Rosignoli C., Alborali G.L. (2019). Detection of a New Genetic Cluster of Influenza D Virus in Italian Cattle. Viruses.

[35] Dane H., Duffy C., Guelbenzu M., Hause B., Fee S., Forster F., McMenamy M.J., Lemon K. (2019). Detection of Influenza D Virus in Bovine Respiratory Disease Samples, UK. Transbound. Emerg. Dis..

[36] Flynn O., Gallagher C., Mooney J., Irvine C., Ducatez M., Hause B., McGrath G., Ryan E. (2018). Influenza D Virus in Cattle, Ireland. Emerg. Infect. Dis..

[37] Studer E., Schönecker L., Meylan M., Stucki D., Dijkman R., Holwerda M., Glaus A., Becker J. (2021). Prevalence of BRD-related Viral Pathogens in the Upper Respiratory Tract of Swiss Veal Calves. Animals.

[38] Lim E.H., Lim S.I., Kim M.J., Kwon M.J., Kim M.J., Lee K.B., Choe S.E., An D.J., Hyun B.H., Park J.Y. (2023). First Detection of Influenza D Virus Infection in Cattle and Pigs in the Republic of Korea. Microorganisms.

[39] Anjorin A.A., Moronkeji G.O., Temenu G.O., Maiyegun O.A., Fakorede C.O., Ajoseh S.O., Salami W.O., Abegunrin R.O., Amisu K.O., Akinyemi K.O. (2023). First Molecular Detection of Influenza D Virus in Cattle from Commercial Farm in Nigeria, Sub-Saharan Africa. One Health Bull..

[40] Chiapponi C., Faccini S., De Mattia A., Baioni L., Barbieri I., Rosignoli C., Nigrelli A., Foni E. (2016). Detection of Influenza D Virus among Swine and Cattle, Italy. Emerg. Infect. Dis..

[41] Mazzetto E., Bortolami A., Fusaro A., Mazzacan E., Maniero S., Vascellari M., Beato M.S., Schiavon E., Chiapponi C., Terregino C. (2020). Replication of Influenza D Viruses of Bovine and Swine Origin in Ovine Respiratory Explants and Their Attachment to the Respiratory Tract of Bovine, Sheep, Goat, Horse, and Swine. Front. Microbiol..

[42] Goecke N.B., Liang Y., Otten N.D., Hjulsager C.K., Larsen L.E. (2022). Characterization of Influenza D Virus in Danish Calves. Viruses.

[43] Sreenivasan C., Thomas M., Sheng Z., Hause B.M., Collin E.A., Knudsen D.E., Pillatzki A., Nelson E., Wang D., Kaushik R.S. (2015). Replication and Transmission of the Novel Bovine Influenza D Virus in a Guinea Pig Model. J. Virol..

[44] Murakami S., Endoh M., Kobayashi T., Takenaka-Uema A., Chambers J.K., Uchida K., Nishihara M., Hause B., Horimoto T. (2016). Influenza d virus infection in herd of cattle, Japan. Emerg. Infect. Dis..

[45] Foni E., Chiapponi C., Baioni L., Zanni I., Merenda M., Rosignoli C., Kyriakis C.S., Luini M.V., Mandola M.L., Bolzoni L. (2017). Influenza D in Italy: Towards a Better Understanding of an Emerging Viral Infection in Swine. Sci. Rep..

[46] Jiang W.M., Wang S.C., Peng C., Yu J.M., Zhuang Q.Y., Hou G.Y., Liu S., Li J.P., Chen J.M. (2014). Identification of a Potential Novel Type of Influenza Virus in Bovine in China. Virus Genes.

[47] Mekata H., Yamamoto M., Hamabe S., Tanaka H., Omatsu T., Mizutani T., Hause B.M., Okabayashi T. (2018). Molecular Epidemiological Survey and Phylogenetic Analysis of Bovine Influenza D Virus in Japan. Transbound. Emerg. Dis..

[48] Sreenivasan C.C., Uprety T., Reedy S.E., Temeeyasen G., Hause B.M., Wang D., Li F., Chambers T.M. (2022). Experimental Infection of Horses with Influenza D Virus. Viruses.

[49] Muhammad A.Z., Ali M., Muhammad K., Syed E.U.H., Aman K.U., Waqas A., Hassan J., Muhammad F., Ahmad Q.Y., Khizer A.H.M. (2021). Peeping into the Emerging Threat of Novel Influenza D Virus: A Review. Agric. Rev..

[50] Silveira S., Falkenberg S.M., Kaplan B.S., Crossley B., Ridpath J.F., Bauermann F.B., Fossler C.P., Dargatz D.A., Dassanayake R.P., Vincent A.L. (2019). Serosurvey for Influenza D Virus Exposure in Cattle, United States, 2014-2015. Emerg. Infect. Dis..

[51] Alvarez I.J., Fort M., Pasucci J., Moreno F., Gimenez H., Näslund K., Hägglund S., Zohari S., Valarcher J.F. (2020). Seroprevalence of Influenza D Virus in Bulls in Argentina. J. Vet. Diagn. Investig..

[52] Rosignoli C., Faccini S., Merenda M., Chiapponi C., De Mattia A., Bufalo G., Garbarino C., Baioni L., Bolzoni L., Nigrelli A. (2017). Influenza D Virus Infection in Cattle in Italy. Large Anim. Rev..

[53] Snoeck C.J., Oliva J., Pauly M., Losch S., Wildschutz F., Muller C.P., Hübschen J.M., Ducatez M.F. (2018). Influenza D Virus Circulation in Cattle and Swine, Luxembourg, 2012-2016. Emerg. Infect. Dis..

[54] Oliva J., Eichenbaum A., Belin J., Gaudino M., Guillotin J., Alzieu J.P., Nicollet P., Brugidou R., Gueneau E., Michel E. (2019). Serological Evidence of Influenza D Virus Circulation Among Cattle and Small Ruminants in France. Viruses.

[55] Alvarez I., Hägglund S., Näslund K., Eriksson A., Ahlgren E., Ohlson A., Ducatez M.F., Meyer G., Valarcher J.F., Zohari S. (2023). Detection of Influenza D-Specific Antibodies in Bulk Tank Milk from Swedish Dairy Farms. Viruses.

[56] Kwaśnik M., Rola J., Larska M., Rożek W. (2025). Serological Evidence of Influenza D Virus Circulation among Cattle in Poland. J. Vet. Res..

[57] Horimoto T., Hiono T., Mekata H., Odagiri T., Lei Z., Kobayashi T., Norimine J., Inoshima Y., Hikono H., Murakami K. (2016). Nationwide Distribution of Bovine Influenza D Virus Infection in Japan. PLoS ONE.

[58] Salem E., Cook E.A.J., Lbacha H.A., Oliva J., Awoume F., Aplogan G.L., Hymann E.C., Muloi D., Deem S.L., Alali S. (2017). Serologic Evidence for Influenza C and D Virus among Ruminants and Camelids, Africa, 1991–2015. Emerg. Infect. Dis..

[59] Lion A., Secula A., Rançon C., Boulesteix O., Pinard A., Deslis A., Hägglund S., Salem E., Cassard H., Näslund K. (2021). Enhanced Pathogenesis Caused by Influenza D Virus and Mycoplasma bovis Coinfection in Calves: A Disease Severity Linked with Overexpression of IFN-γ as a Key Player of the Enhanced Innate Immune Response in Lungs. Microbiol. Spectr..

[60] Gorin S., Richard G., Hervé S., Eveno E., Blanchard Y., Jardin A., Paboeuf F., Cherbonnel M., André S., Guérin J.L. (2024). Characterization of Influenza D Virus Reassortant Strain in Swine from Mixed Pig and Beef Farm, France. Emerg. Infect. Dis..

[61] Sekine W., Katayama M., Ohira K., Ichikawa A., Wakabayashi Y., Takenaka-Uema A., Murakami S., Horimoto T. (2026). A Serological Survey of Influenza D Virus Infection in Cattle in Hokkaido, Japan. J. Vet. Med. Sci..

[62] Sanogo I.N., Kouakou C., Batawui K., Djegui F., Byarugaba D.K., Adjin R., Adjabli K., Wabwire-Mangen F., Erima B., Atim G. (2021). Serological Surveillance of Influenza D Virus in Ruminants and Swine in West and East Africa, 2017–2020. Viruses.

