# Supplementary figures and images for: Molecular Characterisation, Isolation, and Antibody Response to Influenza D Virus in Naturally Infected Cattle
Source: Viruses. 2026 May 29;18(6):626. doi: 10.3390/v18060626 (PMC13307713; doi:10.3390/v18060626)

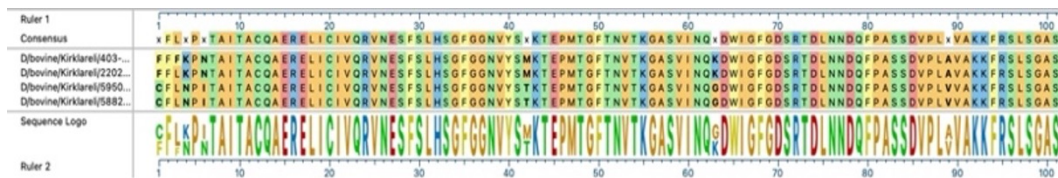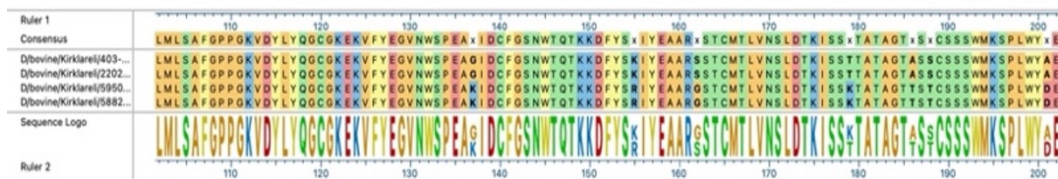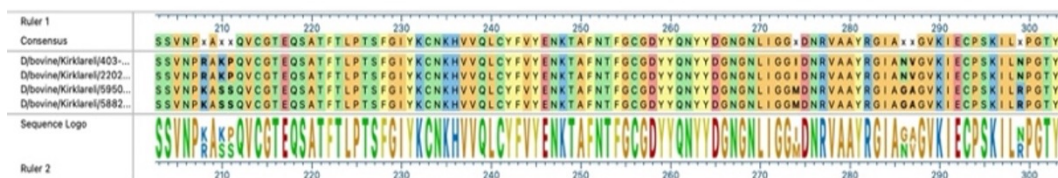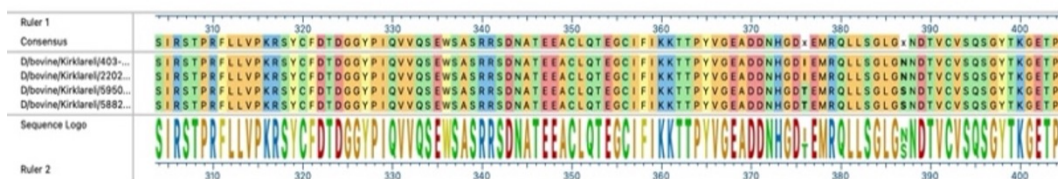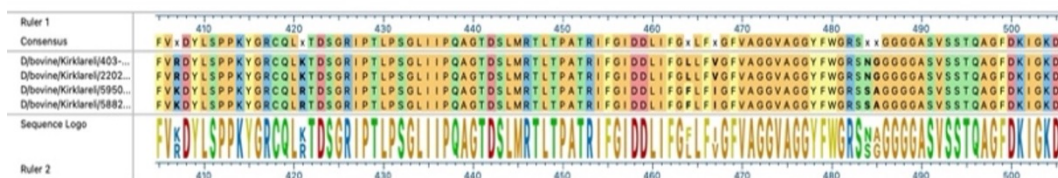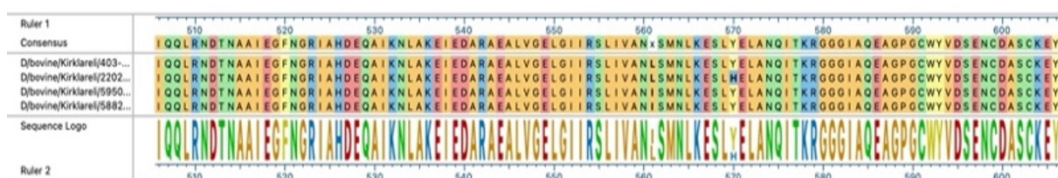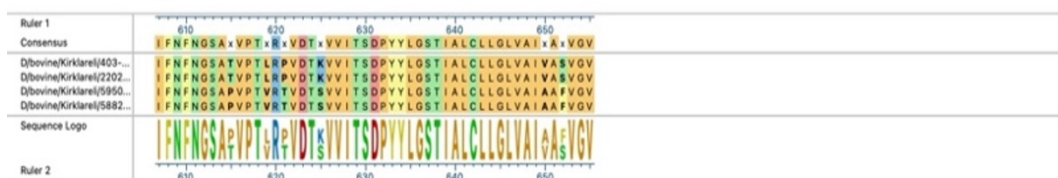

Supplement: Supplementary file 1 [file viruses-18-00626-s001.zip › viruses-4307637-supplementary.pdf]
